# 血小板糖蛋白Ⅰbα抗体IgG1和IgG2a亚型在诱导血小板清除中的作用

**DOI:** 10.3760/cma.j.cn121090-20240710-00256

**Published:** 2025-06

**Authors:** 赛 张, 月 夏, 红磊 叶, 康熙 周, 成林 孙, 梦醒 陈, 克胜 戴, 荣 闫

**Affiliations:** 苏州大学医学院，苏州大学附属第一医院，江苏省血液研究所，国家卫健委血栓与止血重点实验室，苏州 215006 Medical College of Soochow University, Jiangsu Provincial Institute of Hematology; Key Laboratory of Thrombosis and Hemostasis, Ministry of health, Suzhou 215006, China

**Keywords:** 血小板糖蛋白Ⅰbα, IgG2a, 血小板减少, Glycoprotein Ⅰbα, IgG2a, Thrombocytopenia

## Abstract

**目的:**

研究血小板糖蛋白（glycoprotein, GP）Ⅰbα抗体亚型IgG1和IgG2a在诱导血小板清除中的作用。

**方法:**

取健康志愿者静脉血并分离血小板，流式细胞术检测抗人GPⅠbα单抗AN51、AK2、HIP1、TM60、VM16d、WM23、SZ2诱导人急性单核细胞白血病细胞（THP-1细胞）吞噬人血小板的情况；流式细胞术检测比较AN51全抗体、F（ab'）_2_和Fab片段诱导THP-1细胞吞噬血小板的情况；将Fc封闭抗体2.4G2或大鼠IgG2a及IgG1分别通过小鼠眼球后静脉丛注射到C57BL/6J小鼠体内，血细胞分析仪检测2.4G2、IgG2a和IgG1对抗小鼠血小板GPⅠbα抗体R300引起的小鼠血小板数减少的影响。

**结果:**

与抗GPⅠbα抗体IgG1亚型相比，IgG2a亚型的抗GPⅠbα抗体（AN51、TM60）诱导THP-1细胞在体外吞噬血小板的作用更加显著（*P*<0.05）。与AN51全抗体不同，AN51 F（ab'）_2_和Fab片段均不能诱导THP-1细胞吞噬血小板（*P*<0.05）。与对照组相比，注射Fc封闭抗体2.4G2后2、4、6 h时能够减轻R300诱导的血小板减少（*P*<0.05）。此外，注射大鼠IgG2a在2、4、6 h时能够减轻R300引起的小鼠体内血小板减少（*P*<0.05）。

**结论:**

IgG2a亚型在抗GPⅠbα抗体诱导的血小板清除中起作用。

## Abstract (en)

**Objective:**

To investigate the role of the IgG subtypes（IgG1 and IgG2a）in anti-glycoprotein（GP）Ⅰbα antibody-induced platelet clearance.

**Methods:**

Venous blood was collected from healthy volunteers, and platelets were separated. The phagocytosis of human platelets by human acute monocytic leukemia cells（THP-1 cells）induced by different anti-GPⅠbα antibodies（AN51, AK2, HIP1, TM60, VM16d, WM23, and SZ2）was detected by flow cytometry. The effects of the AN51 full-length antibody, F（ab'）_2_, and Fab fragments on platelet phagocytosis by THP-1 cells were detected by flow cytometry. Then, the Fc blocking antibody 2.4G2 and normal rat IgG2a or IgG1 were injected into C57BL/6J mice via the posterior ocular vein, and their effects on platelet reduction induced by R300 were detected by a hematology analyzer.

**Results:**

Compared with IgG1, the IgG2a subtype of anti-GPⅠbα antibodies induced the phagocytosis of platelets by THP-1 cells in vitro（*P*<0.05）. In contrast to the AN51 full-length antibody, neither AN51 F（ab'）_2_ nor the Fab fragment could induce THP-1 cells to phagocytose platelets（*P*<0.05）. Compared with the control group, anti-mouse GPⅠbα R300-induced thrombocytopenia in mice was reduced at 2, 4, and 6 h after the injection of Fc blocking antibody 2.4G2（*P*<0.05）. Similarly, R300-induced thrombocytopenia in mice was reduced at 2, 4, and 6 h after the injection of rat IgG2a（*P*<0.05）.

**Conclusion:**

IgG2a plays an important role in anti-GPⅠbα-induced clearance.

原发免疫性血小板减少症（ITP）是一种以血小板过度破坏或生成异常为特征的自身免疫性疾病[1]。临床表现为血小板计数不同程度的减少、皮肤黏膜出血等症状[2]。其致病机制复杂，目前认为免疫调节失衡和抗血小板特异性自身抗体的产生导致血小板清除加快和数量减少[3]–[4]。迄今为止，已被鉴定能够引起ITP的自身免疫性抗体主要有3种：抗胶原蛋白受体（glycoproteins Ⅰa/Ⅱa）抗体[5]、抗糖蛋白（glycoprotein, GP）Ⅱb/Ⅲa抗体和抗GPⅠb-Ⅸ抗体[6]。其中具有GPⅠb-Ⅸ自身抗体ITP患者的血小板减少更为严重且常规治疗效果更差。

GPⅠbα是GPⅠb-Ⅸ复合物的主要亚基，高度糖基化的氮端胞外区域包含多种配体结合位点，包括VWF和凝血酶等[7]。目前，已有多种抗GPⅠbα单克隆抗体被成功开发并广泛应用于血小板相关工作研究。文献报道，利用不同的抗GPⅠbα抗体研究发现，抗GPⅠbα抗体可通过Fc受体非依赖途径引起血小板减少，包括引发GPⅠbα集簇[8]、血小板活化凋亡[9]以及GPⅠbα去唾液酸化等[10]。也有研究发现，注射丙种球蛋白能减轻抗小鼠GPⅠbα抗体p0p4（IgG2b）引起的血小板减少，但没有改善其他亚型引起的血小板减少[11]，提示Fc受体依赖途径也在抗GPⅠbα抗体引起的血小板减少中起作用。由此可见，抗GPⅠbα抗体引起血小板被清除的机制尚未完全阐明。本研究探讨不同抗GPⅠbα抗体亚型在血小板清除中的作用，为治疗含有抗GPⅠbα自身抗体ITP患者提供新思路。

## 材料和方法

1. 试剂与仪器：抗GPⅠbα抗体AK2和钙黄绿素（calcein）均购自英国Abcam公司，HIP1购自美国eBioscience公司，VM16d和HBSS均购自美国Thermo Scientific公司，TM60购自美国LifeSpan BioSciences公司，AN51和SZ2为本实验室制备。AN51 F（ab'）_2_和Fab片段使用美国Thermo Scientific公司生产的IgG Fab和F（ab）_2_片段制备试剂盒生成。大鼠抗小鼠GPⅠbα抗体R300购自德国Emfret公司，大鼠IgG2a、IgG1和PE-抗人CD41抗体购自美国Biolegend公司。2.4G2抗体购自美国Bioscience公司。人急性单核细胞白血病细胞（THP-1细胞）为本实验室保存。PMA购自美国Sigma公司。RPMI 1640培养基购自武汉普诺赛生物有限公司。配制CGS缓冲液、改良的台式缓冲液（MTB）和人或鼠全血抗凝剂（ACD）所用的生化试剂均购自中国苏州科创生物技术有限公司。全自动三分类血液细胞分析仪购自日本Sysmex公司。低速离心机购自中国安徽中科中佳科学仪器有限公司。流式细胞仪（FC500）购自美国Beckman-Coulter公司。

2. 实验动物：实验所用SPF级6～8周纯系雄性C57BL/6J小鼠购自昭衍新药研究有限公司。动物质量合格证号SCXK（苏）2018-0006。在此项研究中，根据中华人民共和国《实验动物管理条例》规定，所有实验动物相关操作均按照《实验动物管理条例》执行，所有小鼠均饲养于苏州大学SPF级动物房内，动物饲养设施合格证号SYXK（苏）2021-0065。

3. 洗涤血小板的分离准备：采集健康人静脉血，按照7∶1的比例用ACD（2.8-柠檬酸三钠，2.2-D-葡萄糖，1.6-柠檬酸）抗凝，轻轻混匀抗凝后的全血，200×*g*离心11 min，得到富血小板血浆（PRP），PRP经1 700×*g*离心2 min，弃去上清液，沉淀部分用CGS缓冲液（0.123 mol/L NaCl，0.033 mol/L D-葡萄糖，0.013 mol/L柠檬酸钠，pH 6.5）重悬，1 500×*g*离心2 min，弃去上清液，沉淀部分重悬于MTB缓冲液（2.5 mmol/L Hepes，150 mmol/L NaCl，2.5 mmol/L KCl，12 mmol/L NaHCO_3_，5.5 mmol/L D-葡萄糖，1 mmol/L CaCl_2_，1 mmol/L MgCl_2_，pH 7.4），调整血小板悬液的浓度至3×10^8^/ml，室温静置1 h备用。健康志愿者全血样本均通过苏州大学附属第一医院医学伦理委员会批准［2017伦审（申报）批第114号］，所有受试者均知情同意。

4. 细胞培养：THP-1细胞使用含10％灭活的胎牛血清、100 U/ml青/链霉素双抗（C125C8，中国NCM Biotech公司产品）RPMI 1640培养基培养（37 °C，含5％ CO_2_，饱和湿度条件），细胞密度达80％～90％时传代，取对数生长期且生长状态良好的细胞进行实验。

5. 抗人血小板GPⅠbα抗体：本研究选取7种常用的抗人血小板GPⅠbα抗体（表1），其中AN51和TM60为IgG2a型，其余均为IgG1型。

**表1:** 抗人血小板糖蛋白Ibα抗体亚型

抗体名称	来源	亚型
AN51	小鼠	IgG2a
SZ2	小鼠	IgG1
AK2	小鼠	IgG1
HIP1	小鼠	IgG1
TM60	小鼠	IgG2a
VM16d	小鼠	IgG1
WM23	小鼠	IgG1

6. 巨噬细胞吞噬血小板的流式细胞术检测：THP-1细胞用TGF-β1（1 ng/ml）和1,25-（OH）_2_-维生素D3（50 nmol/L）分化培养24 h后接种于24孔板（1×10^6^/ml）中，用PMA（15 ng/ml）作用THP-1细胞45 min后，弃上清，每孔加200 µl HBSS（含Ca^2+^、Mg^2+^），再分别加入10 µg/ml IgG、AN51或F（ab'）_2_和Fab片段、AK2、HIP1、TM60、VM16d、WM23或SZ2孵育且用calcein标记的人洗涤血小板，于37 °C共孵育1 h，PBS洗涤后加入0.1％胰酶-EDTA在37 °C下放置5 min，以去除细胞外黏附的血小板。最后加入终浓度5 mmol/L EDTA消化并收集细胞。加入PE标记的抗人CD41抗体，避光反应15 min后，流式细胞术检测THP-1细胞吞噬血小板情况。用流式细胞术收集1万个THP-1细胞［钙黄绿素（FL1）阳性且PE（FL2）阴性的细胞为吞噬血小板的细胞］。

7. 血小板数量检测：提前10 min，用1 ml注射器将2.4G2（0.5 µg/g）、大鼠IgG2a（0.5 µg/g）或大鼠IgG1（0.5 µg/g）通过小鼠眼球后静脉丛注射到小鼠体内，用同样的方式注射抗小鼠血小板抗体R300（0.1 µg/g）。ACD抗凝，在注射后不同时间点（0、0.5、2、4、6和24 h）用毛细吸管经小鼠眼球外眦静脉丛采血，利用血细胞分析仪检测血小板数目变化。

8. 统计学处理：采用SPSS 18.0软件和GraphPad Prism 10.0软件对实验数据进行统计学分析。所有数据以至少3组独立实验的“均数±标准差”表示，多组比较用单因素方差分析或双因素方差分析，*P*<0.05为差异有统计学意义。

## 结果

1. IgG2a型抗GPⅠbα抗体介导吞噬细胞吞噬血小板：为了研究上述抗人血小板GPⅠbα抗体能否引起吞噬细胞吞噬血小板，预先使用不同的抗体AN51、AK2、HIP1、TM60、VM16d、SZ2或WM23在体外处理人的洗涤血小板，然后与THP-1细胞共同孵育后，通过流式细胞术检测吞噬血小板的THP-1细胞（FL1阳性和FL2阴性）比例，结果如图1所示，与正常小鼠IgG对照组［（4.3±4.5）％］及GPⅠbα IgG1亚型抗体处理组［AK2：（5.2±5.3）％；HIP1：（4.5±4.7）％；VM16d：（5.4±5.2）％；SZ2：（3.5±3.1）％；WM23：（4.1±4.2）％）］相比，GPⅠbα IgG2a亚型抗体AN51［（18.3±10.7）％］及TM60［（16.3±6.6）％］处理的血小板与THP-1细胞共反应后，吞噬血小板的THP-1细胞比例显著增加（*P*<0.05），表明IgG2a亚型抗体能够引起吞噬细胞吞噬血小板。

**图1:**
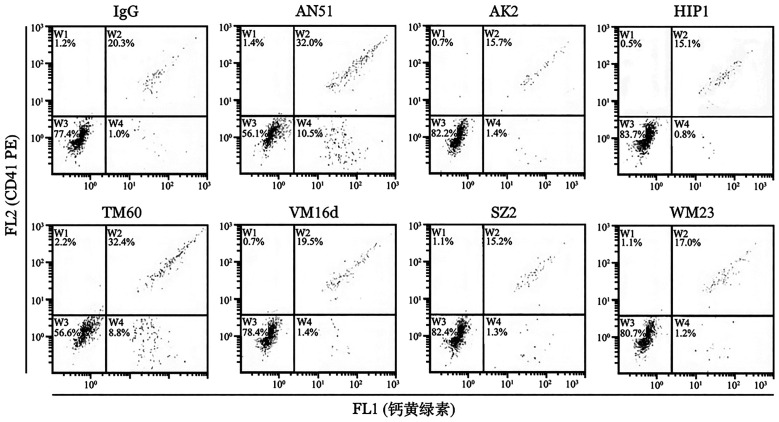
流式细胞术检测抗GPⅠbα单克隆抗体对人急性单核细胞白血病细胞（THP-1细胞）吞噬血小板的影响 **注** 钙黄绿素（FL1）阳性且PE（FL2）阴性的细胞为吞噬血小板的细胞

2. 抗体AN51体外通过Fc部分介导吞噬细胞吞噬人血小板：分别使用AN51的全抗体、F（ab'）_2_和Fab片段在体外处理人的洗涤血小板后，与THP-1细胞共同孵育，通过流式细胞术检测吞噬血小板的THP-1细胞比例，我们发现，与IgG对照组相比，血小板经AN51全抗体处理后，吞噬血小板的THP-1细胞比例显著增加［（21.8±9.8）％对（5.6±4.4）％，*P*<0.05］，而AN51的F（ab'）_2_片段［（4.1±2.3）％对（5.6±4.4）％，*P*>0.05］和Fab片段［（4.2±3.0）％对（5.6±4.4）％，*P*>0.05］处理的血小板不能增加吞噬血小板的THP-1细胞比例。上述结果提示AN51诱导的血小板体外吞噬是由抗体的Fc部分介导的。

3. 封闭Fc受体减轻抗GPIⅠbα抗体导致的血小板减少：预先将Fc封闭抗体2.4G2通过眼眶后静脉丛注射到小鼠体内，然后注射抗小鼠血小板GPⅠbα抗体R300，在注射后0 h（基础值）、2、4、6、24 h眼眶采血检测血小板数量，分别与血小板数量基础值相比得到剩余血小板百分比。注射Fc封闭抗体2.4G2后2、4、6 h时R300处理的小鼠剩余血小板百分比高于对照组小鼠（*P*<0.05）（表2），表明封闭Fc识别受体能够抑制吞噬细胞对血小板的清除，从而减轻抗GPⅠbα抗体导致的血小板减少。

**表2:** Fc受体封闭抗体对抗GPⅠbα抗体R300诱导的小鼠血小板减少的影响（％，*x*±*s*）

组别	样本数	0 h	0.5 h	2 h	4 h	6 h	24 h
R300处理组	6	100±0	69.7±22.9	33.0±25.2	26.0±20.2	26.0±19.9	28.5±14.0
2.4G2+R300处理组	6	100±0	85.3±3.3	58.3±13.4^a^	47.8±14.3^b^	46.8±10.7^b^	34.3±10.6

**注** 表中数值均为不同时间点采血所得到的不同小鼠体内血小板数量占注射前（0 h）各自的基础血小板数量的百分比。与R300处理组相比，^a^*P*<0.01，^b^*P*<0.05

4. 大鼠IgG2a减轻抗GPⅠbα抗体导致的血小板减少：为了探讨抗GPⅠbα抗体IgG2a和IgG1亚型在体内诱导血小板清除的情况，通过眼眶静脉提前10分钟向小鼠体内注射大鼠IgG2a、IgG1或PBS对照，然后再注射抗小鼠血小板GPⅠbα抗体R300清除血小板，在注射前2 h（基础值）和注射后0、2、4、6、24、48 h眼眶采血检测血小板数量，分别与血小板数量基础值相比得到剩余血小板百分比。与R300处理组相比，注射大鼠IgG2a在2、4、6 h增加了R300处理小鼠的剩余血小板百分比（*P*<0.05）；与注射大鼠IgG1相比，注射大鼠IgG2a也增加了4、6 h时R300处理小鼠的剩余血小板百分比（*P*<0.05）；注射大鼠IgG1后，剩余血小板百分比与对照组差异无统计学意义（详见表3）。以上结果提示IgG2a可结合吞噬细胞的Fc受体，从而阻断IgG2a亚型的抗GPⅠbα抗体诱导的血小板清除，减轻R300引起的小鼠血小板减少。

**表3:** 大鼠IgG1和IgG2a对抗GPⅠbα抗体R300诱导的小鼠血小板减少的影响（％，*x*±*s*）

组别	样本数	−2 h	0 h	0.5 h	2 h	4 h	6 h	24 h	48 h
R300处理组	5	100±0	101.5±18.0	91.3±22.5	53.2±5.9	39.1±12.3	30.0±7.36	44.2±17.1	73.4±22.6
IgG1+R300处理组	5	100±0	100.5±13.5	90.4±26.9	71.9±26.7	51.3±14.3	43.6±15.8	48.5±5.5	71.7±7.6
IgG2a+R300处理组	5	100±0	113.8±14.3	108.4±10.5	80.7±12.7^a^	76.8±11.3^a,b^	69.4±14.4^a,b^	65.2±12.5	88.1±8.9

**注** 表中数值为不同时间点采血所得到的不同小鼠体内血小板数量占注射前（−2 h）基础血小板数量的百分比。a：与R300处理组相比*P*<0.05；b：与IgG1+R300处理组相比*P*<0.05

## 讨论

ITP是一种常见的出血性疾病，抗血小板膜表面糖蛋白自身抗体的产生被证明是ITP患者血小板被过度清除的主要因素。临床研究数据表明多数ITP患者产生针对抗GPⅡbⅢa的自身抗体（整合素αⅡbβ3），少数为抗GPⅠb-Ⅸ抗体[12]。而具有抗GPⅠbα抗体的ITP患者表现出对常规治疗（静脉注射免疫球蛋白、糖皮质激素和TPO模拟物[13]等）反应较差，甚至脾切除术都难以治愈。近年来的研究显示，抗GPⅠbα抗体通过Fc受体非依赖途径引起血小板减低。尽管GPⅠbα集簇或去唾液酸化抑制剂能够明显减轻抗GPⅠbα抗体引起的血小板减少，但未能完全抑制[8]。在本研究中，我们发现抗GPⅠbα抗体仍然可通过Fc受体依赖途径导致血小板被吞噬和清除，而且，与IgG1型抗GPⅠbα抗体相比，IgG2a型抗GPⅠbα抗体有更强的促吞噬效果，这与不同的IgG亚型对Fc受体的亲和力不同相一致。IgG1对抑制性Fc受体具有更高的亲和力，而IgG2a和IgG2b对Fc受体具有更高的亲和力[14]–[15]。IgG不同亚型已被证明与FcγR的不同亲和力决定了它们在体内不同的保护和致病活性，FcγRⅠ以高亲和力选择性地与IgG2a结合，FcγRⅣ以中等亲和力与IgG2a和Ig2b结合，而FcγRⅡB和FcγRⅢ以低亲和力与IgG1、IgG2a和IgG2b结合[14],[16]。由于有很多不同的细胞类型会表达相关的FcγR，具体哪一种在血小板清除的过程中起到最主要的作用还未可知。但是已经有研究发现通过阻断FcγRⅣ与致病性抗血小板抗体的结合可以保护小鼠免受抗体诱导的血小板减少，而FcγRⅣ以中等亲和力与IgG2a结合，并不与IgG1相结合[15]。

我们已发表的研究结果显示，AN51通过引起GPⅠbα集簇诱导血小板活化和凋亡，导致血小板通过Fc受体非依赖途径被清除[7]–[8]。本研究显示AN51可在体外诱导THP-1细胞吞噬血小板，而且这种吞噬作用依赖于抗体的Fc段，说明AN51通过Fc受体依赖途径导致血小板被吞噬。因此推测，AN51可能通过Fc受体依赖和非依赖双重途径导致血小板在体内清除，对此我们将在后续研究中进一步探讨。

R300是多种抗小鼠GPⅠbα单克隆抗体的混合物，很好的模拟了ITP患者体内抗GPⅠbα自身抗体的复杂情况。与体外结果一致，用2.4G2封闭Fc受体或者用IgG2a竞争性结合Fc受体，均可减轻R300诱导的小鼠血小板减少，提示抗GPⅠbα抗体在体内同样存在Fc受体依赖的清除途径，而且IgG2a亚型在巨噬细胞吞噬血小板导致血小板清除的过程中似乎发挥了主要作用。与此一致的是，有报道表明与抗血小板IgG1亚型抗体相比，向狒狒体内注射IgG2（a和b）亚型抗体会引起更为严重的血小板减少[17]。IgG2a亚型可能通过与巨噬细胞Fc受体表现出更强的亲和力从而有效介导了抗体诱导的吞噬作用。Chan等[18]研究了ITP患者中抗GPⅡbⅢa自身抗体的亚型分布及与疾病严重程度的相关性，与IgG1相比，IgG2亚型的存在似乎与更低的血小板数目和更难治愈相关。抗体亚型与ITP（尤其是含有GPⅠbα自身抗体）患者疾病程度的关系还需进一步研究。

综上所述，本研究利用细胞吞噬实验和被动型ITP小鼠模型证实IgG2a型抗GPⅠbα抗体在诱导血小板被吞噬和清除中的重要作用，提示不同自身抗体亚型在含GPⅠbα自身抗体的ITP患者中可能发挥不同的作用。因此使用特异亚型IgG可能具有更为有效的封闭效果，为此类ITP患者提供更为有效的治疗思路。
